# Effect of two lipid-lowering strategies on high-density lipoprotein function and some HDL-related proteins: a randomized clinical trial

**DOI:** 10.1186/s12944-017-0433-6

**Published:** 2017-02-28

**Authors:** Chan Joo Lee, Seungbum Choi, Dong Huey Cheon, Kyeong Yeon Kim, Eun Jeong Cheon, Soo-jin Ann, Hye-Min Noh, Sungha Park, Seok-Min Kang, Donghoon Choi, Ji Eun Lee, Sang-Hak Lee

**Affiliations:** 1 Division of Cardiology, Department of Internal Medicine, Severance Hospital, Yonsei University College of Medicine, Seoul, Korea; 2 Cardiovascular Research Institute, Yonsei University College of Medicine, Seoul, Korea; 3 Gachon Cardiovascular Research Institute, Gachon University, Incheon, Korea; 4 Center for Theragnosis, Biomedical Research Institute, Korea Institute of Science and Technology, Seoul, Korea; 5 Interdisciplinary program of Integrated Biotechnology, Sogang University, Seoul, Korea; 6 Department of chemistry, Sookmyung Women’s University, Seoul, Korea

**Keywords:** Atorvastatin calcium, Ezetimibe, Cholesterol-efflux regulatory protein, Inflammation

## Abstract

**Background:**

The influence of lipid-lowering therapy on high-density lipoprotein (HDL) is incompletely understood. We compared the effect of two lipid-lowering strategies on HDL functions and identified some HDL-related proteins.

**Methods:**

Thirty two patients were initially screened and HDLs of 21 patients were finally analyzed. Patients were randomized to receive atorvastatin 20 mg (*n* = 11) or atorvastatin 5 mg/ezetimibe 10 mg combination (*n* = 10) for 8 weeks. The cholesterol efflux capacity and other anti-inflammatory functions were assessed based on HDLs of the participants before and after treatment. Pre-specified HDL proteins of the same HDL samples were measured.

**Results:**

The post-treatment increase in cholesterol efflux capacities was similar between the groups (35.6% and 34.6% for mono-therapy and combination, respectively, *p* = 0.60). Changes in nitric oxide (NO) production, vascular cell adhesion molecule-1 (VCAM-1) expression, and reactive oxygen species (ROS) production were similar between the groups. The baseline cholesterol efflux capacity correlated positively with apolipoprotein (apo)A1 and C3, whereas apoA1 and apoC1 showed inverse associations with VCAM-1 expression. The changes in the cholesterol efflux capacity were positively correlated with multiple HDL proteins, especially apoA2.

**Conclusions:**

Two regimens increased the cholesterol efflux capacity of HDL comparably. Multiple HDL proteins, not limited to apoA1, showed a correlation with HDL functions. These results indicate that conventional lipid therapy may have additional effects on HDL functions with changes in HDL proteins.

**Trial registration:**

ClinicalTrials.gov, number NCT02942602.

**Electronic supplementary material:**

The online version of this article (doi:10.1186/s12944-017-0433-6) contains supplementary material, which is available to authorized users.

## Background

The role of high-density lipoprotein (HDL) in vascular disease is under active investigation. For instance, the cholesterol efflux capacity was reported to be inversely associated with the incidence of cardiovascular events [1]. However, the clinical implication and role of the HDL function are not yet fully established. On the other hand, lowering the low-density lipoprotein-cholesterol (LDL-C) using statins has been the mainstay of pharmacologic therapy aimed at effectively reducing cardiovascular risk [2]. Therefore, most of latest guidelines on lipid management have adopted statins as first-line agents [3, 4]. In a recent IMPROVE-IT trial, the application of simvastatin 40 mg/ezetimibe 10 mg combination was shown to reduce cardiovascular risk compared to using simvastatin 40 mg alone [5]. The addition of ezetimibe 10 mg to ongoing statin therapy is highly effective in LDL-C-lowering. However, whether the combination of ezetimibe/statin has differential pleiotropic effect, such as modification of HDL function, compared to higher dose statin is not yet completely understood.

The progress in analytical methodology has enabled the identification of diverse proteins comprising HDL, and the list continues to grow. Although traditionally known to act in lipid transport, numerous HDL proteins now appear to be involved in other biological functions such as the acute phase response [6] and inflammation [7]. The differential expression of proteins including apolipoprotein (apo)C3 in a diseased condition showed alteration in other HDL function [8]. Modification of HDL particles or their proteins is supposed to influence HDL function. For instance, oxidative stress and inflammation are reported to change the composition of proteins and function of HDL [9]. However, current medical treatment is insufficient to inhibit atherosclerotic process related to dysfunctional HDL. Therefore, further studies of treatment focusing on HDL function would be recommended. To date, it has been reported that HDL-related proteins such as apolipoprotein E [10], paraoxonase-1 [11] or alpha-1 antitrypsin [12] could be affected by lipid-lowering drugs.

The aim of this study was to compare the effects of two lipid-lowering strategies, atorvastatin 20 mg and atorvastatin 5 mg/ezetimibe 10 mg combination, on HDL functions. Therefore, we examined the influence of the drugs on HDL: the cholesterol efflux capacity, endothelial nitric oxide (NO) production, vascular cell adhesion molecule-1 (VCAM-1) expression, and production of reactive oxygen species (ROS) by macrophages. Additionally, we attempted to identify the specific HDL proteins associated with each function. Particularly, we measured apoA1, apoA2, apoC1, apoC2, and apoC3. We also examined whether the changes in the HDL functions correlate with altered HDL proteins following drug treatment.

## Methods

### Study population

Patients who met the 2013 American College of Cardiology/American Heart Association criteria for receiving lipid-lowering therapy were eligible for this study. They consisted of patients who had a prior history of atherosclerotic cardiovascular disease, those who had diabetes mellitus, or high cardiovascular risk. The patients were statin-naïve or had discontinued any lipid-lowering agent at least for 3 months before the enrollment unless they had previous cardiovascular disease. Patients were excluded if they were pregnant or breast feeding, had a history of acute cardio- or cerebro-vascular disease within 3 months before the study, uncontrolled hypertension or diabetes mellitus, thyroid dysfunction, serum transaminase >2 times the upper limit of normal, serum creatinine >1.5 mg/dL, an acute or chronic infection or inflammation, or a history of cancer or adverse events associated with test drugs including myopathy. All the patients provided written informed consent.

### Study protocol

The present study was a sub-study of an 8-week, randomized, open-label interventional study that was approved by Yonsei University Health System, Severance Hospital, Institutional Review Board (4-2013-0281). This study was actually a sub-study of a main trial as mentioned in the methods section, and we applied the clinical trial protocol that was used in the main study. Since our trial followed the protocol, which was revised after the study began, we included the flowchart of revised version of the protocol (Additional file 1: Figure S1). At the initial screening visit, the patients were interviewed to obtain their medical histories and then underwent laboratory assessments. Those who met the criteria for lipid-lowering therapy were subsequently randomized in a 1:1 ratio into two treatment groups for 8 weeks: atorvastatin 20 mg (Lipitor, Pfizer, New York, NY, USA) or atorvastatin/ezetimibe 5 mg/10 mg (Lipitor, Pfizer and Ezetrol, Merck & Co., Whitehouse Station, NJ, USA). These two regimens were selected on our previous studies that showed these two regimens reduced LDL-C levels similarly [13, 14]. Thirty-two patients were initially screened but 3 of them did not complete the study: 2 due to refuse to follow-up and 1 due to protocol violation. Other eight patients were excluded due to insufficient blood sampling. Because of high censoring rate in the atorvastatin monotherapy group, enrollment with unequal allocation was performed to avoid decreasing the power of the comparison. HDL function and proteins were finally analyzed in 21 patients (11 in the atorvastatin monotherapy group and 10 in the combination group; Additional file 1: Figure S1).

### Blood sampling and isolation of HDL

Blood samples were collected from the patients on enrollment and after the 8-week drug treatment. The patients were instructed to fast and avoid alcohol beverages or smoking for at least 12 h before the collection of the samples, which were analysed within 4 h. All the analyses were performed by a local laboratory, certified by the Korean Society of Laboratory Medicine. The lipid levels were measured using an auto-analyzer.

HDL was isolated by ultracentrifugation described below. Briefly, 2 mL of serum sample was transferred into a 12 mL-ultracentrifuge tube (Polyallomer, Beckman Coulter Korea Ltd, Seoul, Korea) and then 0.12 g potassium bromide (KBr) and 0.045 g sucrose were added to be dissolved. Then, 2 mL of solution B (1 mL distilled water, sodium chloride [NaCl] 0.012 g, and KBr 0.135 g), 4 mL of solution A (distilled water 1 mL plus NaCl 0.012 g plus KBr 0.318 g), and 4 mL of distilled water were sequentially added. Ultracentrifugation was conducted using a Beckman Coulter XL-100 K Table Top Ultracentrifuge with a Beckman fixed-angle rotor (SW41Ti) for 18 h at 35,000 rpm. Then, the supernatant, contained the very low-density lipoprotein and LDL was removed and HDL was aspirated. The isolated HDL was subsequently desalted and concentrated with an Amicon 3 k Ultracentrifugal Filter device (Merck Millipore Korea, Seoul, Korea) at 3000 rpm at 4 °C.

### In vitro tests of HDL function

The cholesterol efflux assay was performed using a previously described method [15]. Briefly, the J774 cells were plated and radiolabeled with 2 μCi of ^3^H-choelsterol/mL for 24 h. For the upregulation of adenosine triphosphate (ATP)-binding cassette transporter subfamily member A1 (ABCA1), the cells were incubated with medium containing 0.2% bovine serum albumin (BSA) and 0.3 mM cyclic adenosine monophosphate (cAMP) for 2 h. Then, the medium was changed to a medium containing 0.2% BSA and HDL for 4 h. The experiment was conducted by treatment the cells with acyl-coenzyme A:cholesterol acyltransferase inhibitor 2 μg/mL. The cholesterol efflux proportion was calculated using the following formula: Cholesterol efflux capacity (%) = [^3^H-cholesterol (μCi) in medium containing HDL/(^3^H-cholesterol {μCi} in medium containing HDL + μCi of ^3^H-cholesterol {μCi} in cells)] x 100. The values were adjusted based on the efflux capacity of the pooled serum run in each plate. Each sample was run in duplicate.

The endothelial NO production was assayed as described previously [16, 17]. Briefly, the human umbilical vein endothelial cells were purchased from Lonza (Basel, Switzerland), grown until the cells had reached 90% confluency, and then incubated with serum-free medium overnight. After being treated with 50 μg/mL of HDL, cells were washed and lysed in 5 mM Tris. After centrifuging the cell lysates, supernatants were transferred to Amicon 10 kDa cut-off filter tubes (Merck Millipore Korea) and further centrifuged. Then the flow-through was collected and nitrite level was measured using a kit according to manufacturer’s instruction (Cayman Chemical, Ann Arbor, MI, USA).

The VCAM-1 level was measured by western blotting [18, 19]. Briefly, the human umbilical vein endothelial cells were grown and VCAM-1 expression was induced by 5 ng/mL of tumor necrosis factor-α in serum-free media overnight. Then, the cells were treated with 50 μg/mL of HDL for 4 h, washed, and lysed in radioimmunoprecipitation assay buffer supplemented with protease inhibitor cocktail tablet (Roche Applied Science, Penzberg, Germany). After that, the total protein concentration of cell lysate supernatant was determined, then 7 μg of protein was loaded, and subsequently separated by running the 10% sodium dodecyl sulphate-polyacrylamide gel electrophoresis (SDS-PAGE). The proteins were transferred from the gel to a nitrocellulose membrane and incubated with anti-VCAM-1 (Abcam, Cambridge, MA, USA) and mouse anti-β-actin antibodies (Santa Cruz Biotechnology, Inc., Santa Cruz, CA, USA). Protein bands were visualized using the SuperSignal West Pico Chemiluminescent substrate (ThermoFisher Scientific, Waltham, MA, USA), and the band intensity was quantified using the ImageJ software (National Institute of Health, Bethesda, MD, USA). The VCAM-1 expression was normalized to the intensity of β–actin, and the levels in cells treated with each HDL sample were presented as percentages of untreated cells.

The generation of intracellular ROS was determined using dichlorodihydrofluorescein diacetate (CM-H2DCFDA, ThermoFisher Scientific) [20, 21]. After treating J774 cells with 100 μg/mL of HDL for 24 h, they were stained with 5 μM CM-H2DCFDA in PBS for 24 min at 37 °C, incubated with or without 100 M hydroperoxide for 20 min, and then ROS generation was detected using a flow cytometer. The mean fluorescence intensity was measured in 10,000 cells using the fluorescein isothiocyanate channel.

### Measurement of selected HDL proteins

From preliminary proteomic analysis of HDL samples based on the protocol we previously reported [22], we selected five HDL-related proteins that were abundantly and reproducibly detected: apoA1, apoA2, apoC1, poC2, and apoC3. The proteins were measured and quantified as follows. Briefly, the same amount (from 0.5 to 10 μg) of the HDL samples were separated by 15% SDS-PAGE and transferred onto polyvinylidene difluoride membranes, which were blocked against nonspecific binding, and then incubated with primary antibodies against apoA1, apoA2 (Santa Cruz Biotechnology), apoC1, apoC2 (Abcam), and apoC3 (Academy Bio-Medical Company Inc., Houston, TX, USA). Then, the membranes were further incubated with appropriate horseradish peroxidase-conjugated bovine anti-mouse or goat anti-rabbit secondary antibodies (Santa Cruz). The signal was detected using chemiluminescence with ECL reagent (GE Healthcare, Piscataway, NJ, USA) and the band intensities were quantified using the ImageJ software.

### Statistical analysis

The clinical and laboratory variables were compared by the Student’s *t*-test or chi-square test. A paired *t*-test was used to compare the parameters before and after drug treatment. For the variables showing a skewed distribution, the Wilcoxon signed-rank test for the median was used. The Spearman correlation analysis was used to evaluate the association between the HDL functional parameters and HDL-related protein levels as well as the changes of HDL function and those of HDL protein levels. All the analyses used two-tailed tests with a significance level of 0.05. The statistics for the social sciences version 17.0 software (SPSS Inc, Chicago, IL, USA) was used for the analyses. This study is registered with ClinicalTrials.gov, number NCT02942602.

## Results

### Clinical characteristics and laboratory values

The median age of the study patients was 57 years, and 18 (85%) were males, while three and 13 (14 and 62%, respectively) had diabetes and coronary artery disease, respectively. The baseline high-density lipoprotein-cholesterol (HDL-C) levels were marginally higher in the atorvastatin group than they were in the combination group (45 mg/dL and 39 mg/dL, respectively, *p* = 0.06). The other clinical and laboratory characteristics were comparable between both groups (Table 1). After the 8-week drug treatment, the LDL-C levels were reduced in both groups, and the median percentage changes were comparable (-31.6% and -31.1%, respectively, *p* = 0.57). Although the HDL-C increased significantly only in the atorvastatin monotherapy group, the median percentage change in the HDL-C did not differ between the two groups (11.5% and 8.1%, respectively, *p* = 0.62, Additional file 1: Table S1).

**Table 1:** Clinical characteristics of the study subjects

	Atorvastatin (*N* = 11)	Combination (*N* = 10)	*p*
Age, years	58 (51, 64)	57 (43, 63)	0.75
Male	9 (82)	9 (90)	1.00
Medical history			
Hypertension	8 (73)	6 (60)	0.88
Diabetes mellitus	2 (18)	1 (10)	1.00
LDL-C ≥160 mg/dL	4 (36)	0 (0)	0.12
Smoking	7 (64)	6 (60)	1.00
Coronary artery disease	6 (55)	7 (70)	0.78
Body mass index, kg/m^2^	24.4 (23.4, 25.6)	25.9 (23.4, 26.5)	0.44
Laboratory values, mg/dL			
Total cholesterol	214 (191, 261)	200 (163, 211)	0.17
Triglyceride	187 (125, 341)	120 (105, 184)	0.26
HDL-C	45 (42, 54)	39 (37, 45)	0.06
LDL-C	110 (95, 155)	134 (102, 142)	0.83
Medications			
Aspirin	7 (64)	7 (70)	1.00
β-blockers	5 (46)	4 (40)	1.00
Calcium channel blockers	3 (27)	3 (30)	1.00
RAS inhibitors	6 (55)	5 (50)	1.00

Variables are expressed as median (25^th^ percentile, 75^th^ percentile) or number (%), *HDL-C* high-density lipoprotein-cholesterol, *LDL-C* low-density lipoprotein-cholesterol, *RAS* renin-angiotensin system

### Changes in HDL function after drug treatment

**Fig. 1:**
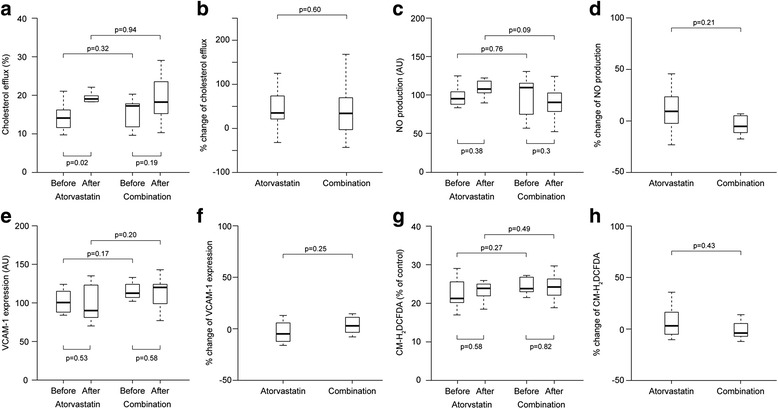
The parameters of high-density lipoprotein (HDL) function and the percentage changes after drug treatment. **a** and **b**) Cholesterol efflux capacity, **c** and **d** nitric oxide (NO) production, **e** and **f** vascular cell adhesion molecule-1 (VCAM-1) expression, and **g** and **h** reactive oxygen species (ROS) production

The baseline median cholesterol efflux capacities were similar between the atorvastatin and combination groups (13.1% and 16.4%, respectively, *p* = 0.32) (Fig 1a). NO and ROS production, as well as VCAM-1 expression, did not differ between the two groups (Figs 1c, e, and g). After the 8-week drug treatment, the cholesterol efflux capacity significantly increased in the atorvastatin, but not in the combination group (Fig 1a). However, the percentage change in the capacities did not differ between the groups (35.6% and 34.6%, respectively, *p* = 0.60, Fig 1b). The NO production did not change significantly after treatment in the monotherapy and combination groups (Fig 1c), and there were no inter-group difference in the percentage changes (9.5% and -5.2%, respectively, *p* = 0.21, Fig 1d). Furthermore, both groups exhibited no changes in the VCAM-1 expressions and ROS production after drug treatment (Figs 1e and g). The percentage changes in the two functional parameters were similar between the monotherapy and combination groups (-5.5% and 2.4%, VCAM-1 expression, *p* = 0.25; 3.0% and -7.2%, ROS production, *p* = 0.43, respectively, Figs 1f and h).

### Relationship between changes in HDL function and HDL-related proteins

The baseline cholesterol efflux capacities were positively correlated with the apoA1 levels (rho = 0.52, *p* = 0.02) and apoC3 (rho = 0.49, *p* = 0.03), but not with the other HDL proteins. ApoA1 (rho = -0.73, *p* = 0.001) and apoC1 (rho = -0.53, *p* = 0.02) showed negative associations with the VCAM-1 expression (Table 2), while no proteins revealed any correlation with NO or ROS production.

**Table 2:** Correlation between the parameters of high-density lipoprotein (HDL) function and selected HDL-related proteins

	Cholesterol efflux		NO production		VCAM-1 expression		ROS production	
	rho	p	rho	p	rho	p	rho	p
ApoA1	0.52	0.02	-0.10	0.71	-0.73	0.001	0.26	0.35
ApoA2	-0.10	0.67	0.20	0.48	0.14	0.58	0.05	0.85
ApoC1	0.41	0.73	0.14	0.61	-0.53	0.02	0.01	0.98
ApoC2	0.26	0.28	0.08	0.77	-0.38	0.12	-0.09	0.76
ApoC3	0.49	0.03	-0.05	0.86	-0.40	0.10	-0.04	0.89

*NO* nitric oxide, *VCAM* vascular cell adhesion molecule, *ROS* reactive oxygen species

Furthermore, drug treatment did not induce significant changes in all the investigated HDL proteins, while percentage changes in expression were similar between the two groups for each protein (Additional file 1: Figure S2). However, the change in the cholesterol efflux capacity had a positive correlation with those of apoA1, apoA2, apoC1, apoC2, and apoC3. In particular, the correlation between the changes in capacity and apoA2 was most obvious (rho = 0.75, *p* < 0.001). No association was found between the changes in NO production, VCAM-1 expression, or ROS production and those of each HDL protein (Table 3).

**Table 3:** Correlations between the changes in high-density lipoprotein (HDL) function and those of HDL-related proteins

	Cholesterol efflux		NO production		VCAM-1 expression		ROS production	
	rho	p	rho	p	rho	p	rho	p
ApoA1	0.59	0.01	0.09	0.75	-0.31	0.22	-0.22	0.43
ApoA2	0.75	<0.001	0.11	0.70	-0.12	0.63	0.45	0.11
ApoC1	0.60	0.01	0.26	0.34	-0.08	0.76	-0.06	0.82
ApoC2	0.46	0.048	0.41	0.14	-0.32	0.21	0.46	0.10
ApoC3	0.59	0.01	0.31	0.29	-0.32	0.20	0.41	0.15

*NO* nitric oxide, *VCAM* vascular cell adhesion molecule, *ROS* reactive oxygen species

## Discussion

The following were the major findings of the present study. 1) The increase in HDL-dependent cholesterol efflux capacity was similar in the two groups. 2) Both regimens did not change the effect of HDL on NO production, VCAM-1 expression, or ROS production. 3) The cholesterol efflux capacity was positively associated with apoA1 and apoC3, whereas apoA1 and apoC1 revealed a negative correlation with VCAM-1 expression. 4) The change in cholesterol efflux capacity induced by the drug treatment was linked to the changes in multiple HDL proteins, including apoA2. To the best of our knowledge, this study is the first to report the effect of a statin/ezetimibe combination and compare two statin-based regimens on HDL function. Furthermore, the relationship between drug-induced changes in HDL function and proteins shown in our study may provide an insight into the additional pleiotropic effect of the current lipid-lowering therapy.

A few studies have shown that statins can increase HDL-dependent cholesterol efflux capacity, including a 14% with simvastatin 40 mg [23] and by 9% with pitavastatin 2 mg [11]. However, it was recently reported that atorvastatin did not affect cholesterol efflux capacity in studies using the sera of mice [24] or human [25]. The reason for the inconsistency in the statin effect on this HDL function is not clear yet. Interestingly, the cholesterol efflux has been shown to increase in studies with statin-induced increase in HDL-C [11, 23], whereas it decreased when statins lowered HDL-C [24]. In our study, HDL-C was raised, although not significantly, in both groups. However, we used the same concentration of isolated HDL from each subject and, therefore, the drug effect on the HDL concentration might have been minimized by our method, and the changes in HDL function were possibly due to differences in HDL itself.

Ezetimibe has been known to enhance reverse cholesterol transport and faecal cholesterol excretion [26, 27]. Nevertheless, data on the effect of ezetimibe on HDL function including cholesterol efflux capacity is extremely limited. Recently, it was reported in hamsters that ezetimibe did not change the efflux capacity of the serum after the adjustment of HDL-C levels [27]. Combination therapy with atorvastatin/ezetimibe has induced percentage changes in the cholesterol efflux capacity similar to that induced by atorvastatin monotherapy. However, several points have not been clearly understood by our results. 1) It is not clear whether the increased efflux capacity induced by drug treatment is largely caused by a primary effect on HDL or secondary effect induced by changes in the lipid metabolism. 2) Furthermore, if the observed change is due to a direct effect on HDL, we are not certain if this is attributable to the effects of ezetimibe or low-dose atorvastatin.

The two different regimens used in our study did not affect the anti-inflammatory function of HDL, whereas they enhanced the cholesterol efflux. Studies evaluating the relationship between HDL proteins and HDL functions, particularly anti-inflammatory, have been highly limited. These present results are in agreement with those of Triolo et al. [23] who evaluated the effect of simvastatin. Gordon et al. [12] reported that rosuvastatin increased HDL-related α1-antitrypsin that reduces the production of tumor necrosis factor-α. In addition, Green et al. [10] found that statin/niacin combination reduced HDL-related apoE. Meanwhile, Miyamoto-Sasaki et al. [11] revealed that HDL-associated paraoxonase-1 was increased by pitavastatin. Although we did not focus on paraoxonase-1, this enzyme is known to suppress proinflammatory response and ROS production [28, 29], and act as one of the key HDL-related proteins.

To date, insufficient amount of data exists on the effects of the drugs on HDL proteins and their relations to biological function. In the present study, we evaluated the correlations between the changes in HDL functions and proteins and identified some relationships. Above all, the effect of apoA1 on the functions of HDL observed in our study was very similar to previously reported data. ApoA1 is a major HDL-related protein [6], which is known to be critical for cholesterol efflux [30]. Moreover, apoA1 was required for reconstituted HDL to inhibit the expression of cell adhesion molecules [31]. In the present study, we discovered that the changes in apoA2 correlated with drug-induced changes in the cholesterol efflux capacity. It was demonstrated that HDL particles containing apoA2 without apoA1 effectively enhanced the cholesterol efflux [32]. In addition, the ability of free apoA2 to promote cholesterol efflux was also reported [33] while Remaley et al. [34] and Sankaranarayanan et al. [35] showed that apoA2 plays a role in the ABCA1- and ABCG1-mediated cholesterol efflux, respectively. However, the effect of drug treatment on apoA2 and its influence on HDL has not been shown before. Therefore, our results on apoA2 may provide an insight into the additive effect of lipid-modifying agent that is mediated, at least partly, by HDL-related proteins.

ApoC1 was negatively correlated with the endothelial VCAM-1 expression in our study. Studies regarding investigating the biological role of apoC1 have been very limited, and the results were inconsistent. It has been demonstrated that apoC1 increased the lipopolysaccharide-induced inflammation [36]. Conversely, apoC1 was reported to inhibit pro-inflammatory cytokine production in murine immune cells [37]. Interestingly, the inhibitory effect of HDL on LPS-induced inflammation was reduced in lecithin-cholesterol acyltransferase (LCAT)-deficient mice [38]. It is well known that apoC1 activates LCAT, which is needed for HDL maturation [39]. Based on this background, apoC1 may affect vascular inflammation by LCAT activation and HDL maturation. The role of apoC3 in HDL function has not been completely established. Only recently, proteomic analysis of murine HDL identified the correlation between cholesterol efflux capacity and apoC3 [40]. Our study also discovered this association in human HDLs. Although its clinical relevance is not entirely clear currently, this association was also validated by the relationship observed between the changes in apoC3 and those of the cholesterol efflux in our study.

Our study had some potential limitations. First, we suggested the effect of HDL proteins and the correlations between the drug-induced changes in these proteins and functional changes in the HDL based on systemic analyses. However, the mechanism underlying the biological role of HDL proteins in HDL functions may not have been completely elucidated by this study. Future studies on the proteins that determine the drug-induced changes in HDL function may provide further insights. Second, we chose candidate proteins based on abundancy and reproducibility of the measurement. Nevertheless, numerous other proteins that have been currently reported may have additional effects on the HDL function. For instance, we cannot rule out the potential of the anti-inflammatory HDL-associated enzymes, which may have played a specific role in the in vitro tests we performed. Third, the drug effect shown by in vitro experiment may not be extrapolated to in vivo or clinical outcomes. For a more comprehensive understanding, testing the drug effect in both hepatocytes and other peripheral cells might be helpful. Finally, total number of our subjects was not sufficiently large. However, when designing this study, we referred to those of prior reports that showed drugs-induced changes of cholesterol efflux in relatively small groups of patients [12, 13].

## Conclusion

Taken together, these results suggest that atorvastatin monotherapy and the low-dose atorvastatin/ezetimibe combination similarly promoted the HDL-dependent cholesterol efflux capacity. Furthermore, the HDL-related proteins, including but not limited to apoA1, showed correlations with drug-induced changes as well as the baseline function of HDL. Our study indicates that conventional lipid-lowering therapy may have additional pleiotropic effect on HDL function, at least partly, by the changes of HDL proteins.

## Additional file

Additional file 1: Table S1. Changes in lipid profiles after 8-week drug treatment. **Figure S1.** CONSORT 2010 flow diagram. **Figure S2.** Expression of high-density lipoprotein (HDL)-related proteins before and after drug treatment. (A) ApoA-I, (B) ApoA-II, (C) ApoC-I, (D) ApoC-II, (E) ApoC-III. (ZIP 353 kb)

## Abbreviations

ABCA1: Adenosine triphosphate binding cassette transporter subfamily member A1
BSA: Bovine serum albumin
cAMP: cyclic adenosine monophosphate
CM-H2DCFDA: Dichlorodihydrofluorescein diacetate
HDL: High-density lipoprotein
HDL-C: baseline high-density lipoprotein-cholesterol
LDL-C: Low-density lipoprotein-cholesterol
NO: Nitric oxide
ROS: Reactive oxygen species
SDS-PAGE: Sodium dodecyl sulphate-polyacrylamide gel electrophoresis
VCAM-1: Vascular cell adhesion molecule-1
