# Supplementary material for: Effect of two lipid-lowering strategies on high-density lipoprotein function and some HDL-related proteins: a randomized clinical trial
Source: Lipids Health Dis. 2017 Feb 28;16:49. doi: 10.1186/s12944-017-0433-6 (PMC5331745; doi:10.1186/s12944-017-0433-6)
Supplement: Additional file 1: Table S1. — Changes in lipid profiles after 8-week drug treatment. Figure S1. CONSORT 2010 flow diagram. Figure S2. Expression of high-density lipoprotein (HDL)-related proteins before and after drug treatment. (A) ApoA-I, (B) ApoA-II, (C) ApoC-I, (D) ApoC-II, (E) ApoC-III. (ZIP 353 kb) [file 12944_2017_433_MOESM1_ESM.zip › Two LL on HDL_lipids in HD_SI r.docx]

**Effect of Two Lipid-Lowering Strategies on High-Density Lipoprotein Function and Related Proteins**

Chan Joo Lee, Seungbum Choi, Dong Huey Cheon, Kyeong Yeon Kim, Eun Jeong Cheon, Soo-jin Ann, Hye-Min Noh, Sungha Park, Seok-Min Kang, Donghoon Choi, Ji Eun Lee*, Sang-Hak Lee*

**Additional file 1 Table S1. Changes in lipid profiles after 8-week drug treatment**

|  |  | Atorvastatin (N=11) | Combination (N=10) | p^a^ |  |
| --- | --- | --- | --- | --- | --- |
| Total cholesterol | Before | 214 (191, 261) | 200 (163, 211) | 0.17 |  |
|  | After | 158 (129, 182) | 142 (129, 172) | 0.45 |  |
|  | % change | -26.2 (-31.3, -20.8) | -16.9 (-36.3, -14.4) | 0.38 |  |
|  | p^b^ | 0.004 | 0.004 |  |  |
| Triglyceride | Before | 187 (125, 341) | 120 (105, 184) | 0.26 |  |
|  | After | 114 (74, 164) | 116 (86, 183) | 1.00 |  |
|  | % change | -42.6 (-65.0, 3.7) | -18.1 (-34.7, 17.0) | 0.43 |  |
|  | p^b^ | 0.24 | 0.44 |  |  |
| HDL-C | Before | 45 (42, 54) | 39 (37, 45) | 0.06 |  |
|  | After | 54 (44, 59) | 45 (40, 47) | 0.06 |  |
|  | % change | 11.5 (5.2, 18.2) | 8.1 (0, 15.4) | 0.62 |  |
|  | p^b^ | 0.04 | 0.21 |  |  |
| LDL-C | Before | 110 (95, 155) | 134 (102, 142) | 0.83 |  |
|  | After | 77 (57, 95) | 84 (67, 91) | 0.97 |  |
|  | % change | -31.6 (-52.3, -29.5) | -31.1 (-42.3, -25.0) | 0.57 |  |
|  | p^b^ | 0.002 | 0.004 |  |  |
| Variables are expressed as median (25^th^ percentile, 75^th^ percentile); p^a^: comparison between groups; p^b^: comparison in a groups before and after treatment; HDL-C: high-density lipoprotein-cholesterol; LDL-C: low-density lipoprotein-cholesterol | | | | | |

**Additional file 1 Figure S1. CONSORT 2010 flow diagram.**

**Additional file 1 Figure S2. Expression of high-density lipoprotein (HDL)-related proteins before and after drug treatment. (A) ApoA-I, (B) ApoA-II, (C) ApoC-I, (D) ApoC-II, (E) ApoC-III.**
