# Supplementary figures and images for: Effect of two lipid-lowering strategies on high-density lipoprotein function and some HDL-related proteins: a randomized clinical trial
Source: Lipids Health Dis. 2017 Feb 28;16:49. doi: 10.1186/s12944-017-0433-6 (PMC5331745; doi:10.1186/s12944-017-0433-6)

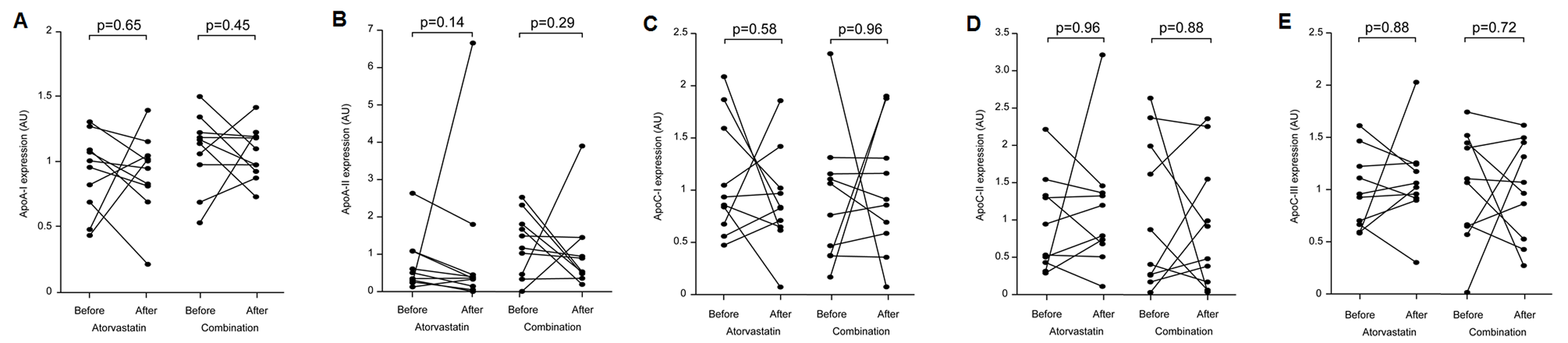

Supplement: Additional file 1: Table S1. — Changes in lipid profiles after 8-week drug treatment. Figure S1. CONSORT 2010 flow diagram. Figure S2. Expression of high-density lipoprotein (HDL)-related proteins before and after drug treatment. (A) ApoA-I, (B) ApoA-II, (C) ApoC-I, (D) ApoC-II, (E) ApoC-III. (ZIP 353 kb) [file 12944_2017_433_MOESM1_ESM.zip › S2_Fig.tif]

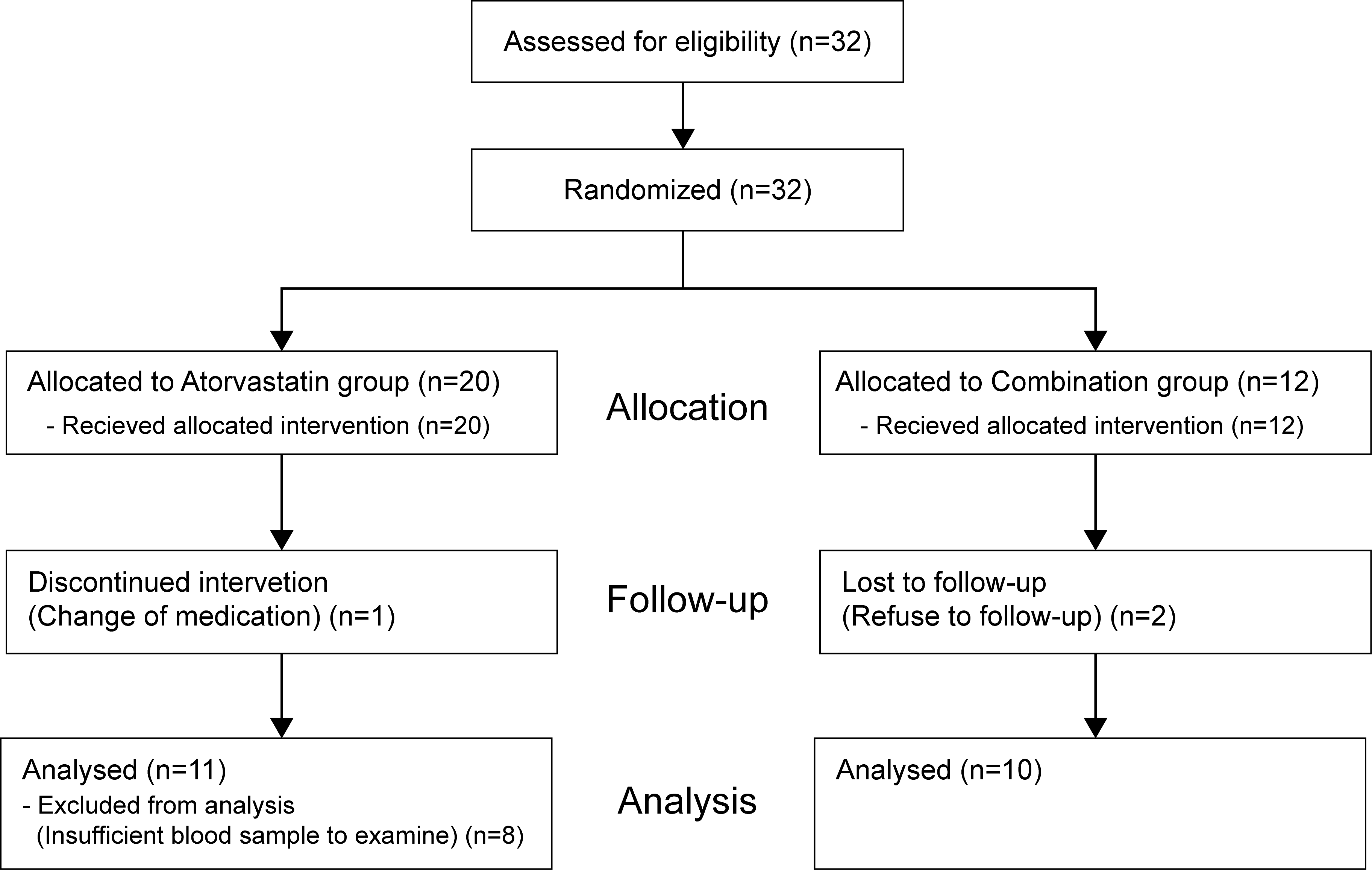

Supplement: Additional file 1: Table S1. — Changes in lipid profiles after 8-week drug treatment. Figure S1. CONSORT 2010 flow diagram. Figure S2. Expression of high-density lipoprotein (HDL)-related proteins before and after drug treatment. (A) ApoA-I, (B) ApoA-II, (C) ApoC-I, (D) ApoC-II, (E) ApoC-III. (ZIP 353 kb) [file 12944_2017_433_MOESM1_ESM.zip › S1_Fig.tif]
